# Report of Forty-Eight Cases of Hospital Gangrene and Hospital Erysipelas Treated in General Hospital, Division No. 1, Charlottesville, Va., from June 1st, 1864 to 1st October, 1864

**Published:** 1864-11

**Authors:** C. R. Harris

**Affiliations:** in charge of Gangrene and Erysipelas Ward.


ART. III.—*Report of Forty-Eight Cases of Hospital Gangrene and Hospital Erysipelas Treated in General Hospital, Division No. 1, Charlottesville, Va., from June 1st,* 1864 *to 1st October,* 1864. By Acting Assistant Surgeon C. R. HARRIS, in charge of Gangrene and Erysipelas Ward.

The gangrene and erysipelas wards in this hospital are a series of well-arranged cloth tents, and are admirably adapted for the important purposes of isolation and æriation.

These diseases are the great scourges of all armies and hospitals. The publication of interesting cases, with their treatment and results, is an imperative duty incumbent upon every member of the medical staff.

We shall therefore present a classified tabular statement or record of cases under treatment, with accompanying results, which we hope may prove of more than ordinary clinical interest to the profession:

| | | |
|---|---|---|
| Total number of cases received in ward from 1st June to 1st October, | | 48 |
| Simple cutaneous erysipelas (traumatic), | | 6 |
| Traumatic phlegmonous erysipelas, | | 9 |
| Hospital gangrene (traumatic), unaccompanied with erysipelas, | | 6 |
| Hospital gangrene complicated with traumatic erysipelas phlegmonodes, | | 25 |
| Idiopathic cutaneous erysipelas, with diarrhœa acute, | | 1 |
| Idiopathic cutaneous erysipelas, with chronic diarrhœa, | | 1 |
| Total, | | 48 |
| Recovered, | 41 | |
| Died, | 7 | |
| | — | 48 |

Of the forty-one cases which recovered, there were eight wounded in and near the elbow joints, upon three of whom the operation of resection was performed. One was wounded at the knee joint, at the same time involving both knee and elbow joints, by a fragment of shell. Three cases of ankle joint and three cases of wrist joints by Minie balls, and one upon whom resection was performed on the ulna for three inches. Total, seventeen cases, involving the important joints of the extremities, all of whom recovered with partial or complete anchylosis, making a total of seventeen cases, one-third of the total number wounded; all of whom were also attacked with either gangrene alone, or complicated with erysipelas phlegmonodes.

Of the seven fatal cases not one died with or from hospital gangrene or erysipelas. Two had compound comminuted fracture of shaft of tibia, in one of whom there was resection of tibia for three inches. They had recovered from gangrene and erysipelas, and the wounds at the seat of fracture were doing well, with a laudable secretion of pus, when they died of puruloid infection or pyæmia. In one there was compound comminuted fracture of humerus at upper third, who, after complete subsidence of gangrene and erysipelas, died of pyæmia, the wound and surrounding tissues looking perfectly healthy. One of the cases, after fully recovering from erysipelas and hospital gangrene, died with his second attack of cholera morbus. The fifth case of strumous habit, and with an enfeebled constitution for years, died of chronic dyspepsia, with anorexia and consequent inanition. The sixth case died of pyæmia—from v. s. of upper third of humerus—producing a severe compound comminuted fracture, involving at the same time the scapula. Several fragments of bone were removed. The wound looked well, and the erysipelatous inflammation had subsided for some time prior to the manifestations of any of the symptoms of puruloid infection. The seventh and last case died also of pyæmia complicated at first with traumatic pneumonia, the result of gun-shot wound of apex of lung and scapula, after the entrance and exit wounds were looking entirely healthy, and the phagedena having subsided for two or three weeks.

The fatal results, therefore, in the cases enumerated were not attributable either to hospital gangrene or traumatic erysipelas. The experience of all hospital surgeons fully corroborate our own experience, that puruloid infection or pyæmia is not confined to wounds of an unhealthy character. We have frequently witnessed patients attacked with this grave and fatal malady, whose stumps were looking well, and whose wounds from projectiles presented all the characteristics belonging to healthy wounds.

All the cases of hospital gangrene enumerated were of a grave character, with the exception of two, and presenting in the aggregate the most destructive sloughing I ever witnessed, embracing in its ravages extensive ranges of tissue, involving the skin, superficial and deep seated fascia, muscular, intermuscular septa and areolar tissue, endangering nerves, arteries, veins and periosteum. A large number of the cases did not come under our treatment until this condition existed, owing, in most instances, to a prejudice on the part of the patients to the infected ward, and also to separating from kind surgeons, matrons, nurses and acquaintances. The opinion, too, was prevalent, to a certain extent, that the removal to the tents was a resting-place on route to the dead-house.

The history, causes and symptoms of hospital gangrene and erysipelas are too familiar to every surgeon, especially those in hospital practice, to demand at our hands a minute description. It is our purpose, therefore, to give an outline of the treatment adopted in connection with foregoing results, and which are divided by all logical writers into local and general or constitutional:

*Local Treatment by Escharotics.*

All surgeons agree in regard to the power and efficacy of escharotics in arresting sloughing, although there exists a difference of opinion in relation to the value and certainty of their favorite remedies, some preferring pure nitric acid and the acid solution of the peroxide of mercury, others the chloride of zinc in combination with the sulphate of copper and lime-water, forming a paste. Our experience, however, with these remedies, has fully satisfied us that they must all yield to the per-sulphate of iron in combination with pure nitric acid or a combination of sulphate of iron with the sulphuric and nitric acids, according to the subjoined formula:

Take—Acid. Sulphuric., 4 drachms; Ferri Sulphas, 4 ounces; Acid. Nitric., 5 drachms; Water, 6 drachms; or this ratio of combination, if you wish a larger amount prepared.

Add the sulphuric acid to the sulphate of iron, stir well, and then pour in the nitric acid to the mixture in a porcelain mortar and stir until fumigation ceases. We find a difference of opinion to exist on the part of the medical staff in relation to the power and efficiency of the per-sulphate of iron, as the mixture is usually termed. This arises from the fact that in moderate cases any preparation, under the strength of the foregoing formula, is too weak to contend with the disease in a large majority of the cases as they occur. We have tried the weaker preparations, and were, in many instances, disappointed. The advantages of this preparation over the pure nitric acid as a safe, efficient, energetic and rapid escharotic are numerous. It affords little or no pain to the surrounding healthy tissue, and much less to the parts diseased, but which are endured with more or less sensibility. This is not the case with the pure nitric acid. It also produces a rapid eschar frequently in two or three applications. Humidity being characteristic of hospital gangrene, the morbid discharge, the result of rapid disintegration, should be washed off. This preparation, however, is not to be thwarted in its action by the fluids. It will require perhaps more frequent applications if this precaution is not observed, yet it will surely produce the desired charr or eschar of the sphacelated tissue, which can then be easily removed or turned out by the handle of the scalpel or spatula. It should never be separated by force.

After the pulpified mass, resembling the parenchyma of the plumb or grape, is thus hardened into a dry eschar or charred, the further use of the escharotic is quite an unnecessary waste of the remedy. The eschar should be removed, if loose and easily separated from the living tissues beneath. The application should be made twice per day until this condition is induced. In order to promote a separation of the dead from the living tissue, we used the tan-oose poultices, procuring the tan-oose from a convenient tan-yard. The decoction is much stronger than that made in the hospitals. We used the decoction from the chesnut oak, the red and spanish oak, ignoring the black oak bark decoction, which, in combination with the per-sulphate of iron, causes a black inky discoloration of the parts, thus preventing a satisfactory inspection by the surgeon. The tanooze poultice proved a most valuable auxiliary by its action upon the surrounding red or livid areolæ which accompanies the phagedena, especially when complicated with cellulo-cutaneous erysipelas. The hardened halo, which presents a true type of the inflammatio debiles, is much improved by the detergent and emollient effects of the oak-bark cataplasm. In addition to these results, the poultice separates the charred and dry from the living tissue, and producing such marked antiseptic properties as to render unnecessary the use in the ward of either Labarraques' or Darby's chlorinated antiseptic mixtures. The oak-bark decoction, composed chiefly of gallic and tannic acids, in addition to its powerful astringent properties, evinced antiseptic qualities of no ordinary power. The tincture of iodine also played an important part in controlling and subduing the deep-seated erysipelatous inflammation of adjacent parts. If the improvement in the constitutional symptoms should not keep pace with the salutary action of the escharotic, after the first eschar is removed, a re-application will become necessary. The skin, cellular tissue, and the areolar elastic fibrous fascia, are the first to yield to the march of the cruel invader. Next the aponeurotic or deep-seated fascia of the limbs, which is so important for forming and enclosing distinct sheaths to important muscles and tendons, is the last to yield. They are easily recognized from the cellular tissue and superficial areolar fibrous tissue by their structure, which is pure white, iridescent and unyielding. The pulpy dead tissue need be touched with the escharotic only, whilst the surgeon can make an effort, frequently successfully, to save the deep-seated fascia; for the redeeming feature of the escharotic is that it injures but slightly, if at all, healthy tissue. Great experience and discretion are required in watching the progress of this grave malady, especially in combination with erysipelas. The coats of the arteries, yielding with great reluctance, must eventually give away, and as we have no solidification or occlusion of their channels up to the collateral branches by plastic lymph, no time is to be lost in arresting the sequelæ of sub-cellular erysipelas as well as the phagedena which will eat and destroy by sloughing and disintegration, the vital and important tissues implicated or threatened.

If, upon the removal of the charred mass of sphacelated tissue, the wound or ulceration should present an unhealthy aspect, with a tendency to a renewal of the sloughing, the escharotic should be again applied, and the poultices applied at each application, which is made with a mop of lint, with the poultices coming immediately in contact with the slough. At each dressing great care should be taken in washing off all sanious pus or ichorous secretions about the parts. If the wound should look healthy and disposed to granulate, evinced by florid sugar-loaf granulations, the simple cold-water dressings are all-sufficient. We sometimes find the diseased parts or hiatus presenting a varied aspect. At one point a small patch of healthy granulations; at another, the true type of the gangrenosa; at a third point, nature is making an effort at reparation by flabby, indolent or exuberant granulations.—We now institute the escharotic and stimulant treatment pro re-nata, using the strong escharotic at one point; the sulphas cupri, 1 drachm, to 1 ounce of water at another; 10 ounces sulph. cupri, 10 grs. to 1 ounce of water at another; thus meeting the varied conditions of the diseased parts, until the whole presents a uniform healthy surface of granulations, followed by reparation and cicatrization, when we again resume the simple dressing.

Burrowing of either ichorous or laudable pus must be sedulously guarded against in erysipelas phlegmonodes, either complicated or not with hospital gangrene, by position and bandages, and with free, deep incisions, to preserve from disorganization the sub-cutaneous and the intermuscular areolar tissue, periosteum and bones, lest your patient shall sink under irritative fever, suppurative chills, and last by puruloid infection or pyæmia. When the disease attacks the extremities, an elevated position greatly aided us with the kind offices of nature to effect a cure and preserve both life and limbs.—In all cases you have an asthenic or hypostatic hyperemia of the limbs. There is a feebleness of nervous energy in the capillary system The blood, like all fluids, will obey the laws of gravitation. By an elevated position, you promote the reparatory process and avert œdema or pitting of the cellular tissue. The low state of vital power is sustained thereby, by observing and controlling (upon the principle of hydrostatics) the ordinary laws of gravitation, thus removing the enfeebled and contaminated nutrition in the surrounding and adjacent tissues, and preventing also the re-accession of the phagedena. We had two or three cases to seriously relapse, who were treated by apposition and alteration from prematurely lowering their limbs, and thus suddenly filling the weakened capillaries in the healthy granulating surfaces, when capillary hemorrhage ensued, with a consequent return of the sloughing. Hence, great vigilance was required in compelling our patients to gradually lower their positions, as they convalesced.

*Constitutional or General Treatment.*

In the beginning, the native causes and predominant type of these diseases should be taken into consideration by the surgeon, as a guide to the adoption of correct principles of therapeutics, upon which all favorable results depend.

A leading characteristic or pathognomic trait of these diseases is an irritative type of typhoid fever, with increased frequency of the pulse, ranging from 100 to 130 beats per minute—with a reduction both in its force and diameter, anxiety of countenance—with involuntary tremors of the muscles of the extremities and the tongue. Upon the admission of our patients, it was frequently the case that they would present all the symptoms attendant upon the excessive use of opium or alcohol, when little or none had been taken. They evinced a striking diminution of the vital forces marked by a perverted change of nerve power, and of the vascular and muscular systems.

The exposure incident to the life of the soldier, his irregularities of diet, in regard to quantity and quality; the wear and tear of marches, and the mental disquietude, are all well calculated to superinduce this depraved and enfeebled condition, thus greatly disturbing the vital functions of circulation and innervation.

The treatment was, therefore, strictly stimulo-tonic and supporting. We endeavored to uphold the vital power of reparation; we invoked also the aid of large calmative doses of opium at bed-time, from one to two drachms of tinct. opii, or four grains of solid opium, which improved the secretions, soothed and quieted the nervous irritation and muttering delirium dependent upon an enfeebled and perverted nutrition of the cerebral mass. We prescribed whiskey in all cases in small doses of one to two ounces every three or four hours; tinct. ferri mur. gtts. xxx, with one gr. quinia as an auxiliary tonic. In cases of erysipelas we gave sulph. quinia 25 to 30 grains every twenty-four hours, in divided doses, in combination, with marked benefit in controlling the chills, irritative fever and profuse and exhausting sweats. Alcohol acted the part of a stimulant to the nervous system; it sustained the process of calorification and shielded the tissues. Alcohol, as a combustible food, is easily absorbed from the blood vessels of the stomach; as a hydro-carbon, in combination with nitrogenous articles of food, it aids in supplying the demands for oxygen to maintain animal heat. It reaches the current of the circulation with great facility, and imposes little or no labor upon either primary or stomach digestion, or secondary digestion. That a deficiency of oxygen in the human system plays the part of cause and effect in relation to hospital gangrene, I do not entertain a doubt. The treatment which we have premised, with accompanying results, will bear us out in the conclusions to which we have arrived.

Since our views and treatment were being written for publication, we have accidentally noticed some written experiments, of more than ordinary interest, made in the Hotel Dieu, in Paris. Dr. Laugier, and also a medical student, treated 2 cases of hospital gangrene in the leg and foot, successfully, by enclosing the diseased parts in an apparatus to disengage oxygen gas continuously. The result was that, in a short time, the gangrene was promptly arrested both in the feet and legs of their patients. Other experiments of like character were performed and followed by similar results, which proved beyond doubt that oxygen was an effectual stimulant or remedy for the disease. I regret that time and space will not now allow me to elaborate fully my views on this important subject. We have designed to give facts, and scarcely more than allude to the therapeutical principles and their application to the grave malady in question.

**Alcohol exerts its influence upon the nutrition of the nervous system** by supplying appropriate fuel or material for the maintenance of animal heat. In small and frequent doses it acts as a specific stimulus to the nervous system, calms and soothes our patients, and as a combustible fluid or food, maintains a normal condition of the nerve cell and tissues. Whilst we are feeding the process of calorification, we are fortifying and guarding, at the same time, against the rapid waste of tissues during the process of oxidation which must occur to maintain it. It is a question of demand and supply. A certain and sure cause of depression of vital power and relapses in three of our patients, was a faulty digestion and anorexia. If our patients did not eat, the gangrene would. The disease would feed upon the tissues until death closed the scene. With the free administration of whiskey our patients took nutritious or nitrogenous food freely and of easy digestion.

In this morbid combination of æsthenic action, growing out of the altered and perverted functions of nutrition and assimilation, there was in most of our cases an impoverished condition of the blood, with a marked diminution of its red corpuscles and also in the quantity of fibrin.

In this condition, the tincture of iron was an indispensable remedy: whilst it improved and aided greatly the powers of digestion, it maintained the desire for appropriate animal food.

Under the treatment described, both local and general, a success more than was reasonably to be expected, rewarded our efforts, and without claiming anything original in the practice, we hope that this paper may be found not without interest to the reader.

